# Editorial: Novel frontiers of retinal neurodegenerative diseases

**DOI:** 10.3389/fmed.2024.1470998

**Published:** 2024-08-14

**Authors:** Wensheng Li, Qinxiang Zheng, Bo Chen, Radouil Tzekov

**Affiliations:** ^1^Shanghai Aier Eye Hospital, Shanghai, China; ^2^Shanghai Aier Eye Institute, Shanghai, China; ^3^Wenzhou Medical University, Wenzhou, China; ^4^Department of Ophthalmology, Icahn School of Medicine at Mount Sinai, New York, NY, United States; ^5^Department of Neuroscience, Icahn School of Medicine at Mount Sinai, New York, NY, United States; ^6^Department of Ophthalmology, University of South Florida, Tampa, FL, United States; ^7^Department of Medical Engineering, University of South Florida, Tampa, FL, United States

**Keywords:** retina, editorial, inherited retinal degenerations, gene therapy, review

Retinal neurodegenerative diseases, such as inherited retinal dystrophies (IRDs), age-related macular degeneration, glaucoma and diabetic retinopathy, represent a significant public health problem worldwide and affect the quality of life for hundreds of millions of people, sometimes leading to permanent vision impairment and blindness. Recent advances in molecular biology and genetics demonstrated that these diseases are highly complex, and their phenotypic expression can be quite variable and, sometimes–poorly understood. Furthermore, few (if any) effective treatments leading to vision restoration and visual function improvement are currently available, especially in treating advanced stage diseases. Therefore, a highly sought-after course of treatment for neuronal loss follows a neuroprotective or regenerative strategy. New cutting-edge technologies, such as gene therapy, stem cell therapy, and tissue engineering are in development. The purpose of this Research Topic was to provide examples for new developments in the diagnosis and treatment of retinal neurodegenerative diseases and summary of some aspects of the field and the potential for future development.

Feng et al. discuss case series of a rare IRD manifesting as an adult-onset neuronal intranuclear inclusion disease. The authors find interesting pigmentary changes involving the macular region and hypofluorescent areas in the peripapillary region, with areas of hyperfluorescence surrounding the hypofluorescent area in some cases. These changes were associated with outer retinal dystrophy and reduced inner retinal thickness on SD-OCT. Furthermore, various functional retinal changes were demonstrated too: decreased pupil size, blind spot enlargement, temporal field defects, ERG and mfERG showed decreased amplitude and increased peak times.

Liu S. et al. report a rare case of a young female patient with a mild form of familial foveal retinoschisis caused by a novel mutation in the CRB1 gene (c.1366 T > C). This case shows an unusual presentation and development, with transitioning from a somewhat typical retinoschisis pattern in the macula to a parafoveal cystoid degeneration with improved visual field sensitivity at an early age of 12 years, much earlier than reported in the literature (typically, age 25–30). Furthermore, the rapid transitioning happened after a head trauma, an unusual occurrence, with an uncertain causative significance.

Yang et al. summarize the phenotype of an early cone-rod dystrophy with macular staphyloma in one individual from a non-inbred Chinese family with two pathogenic missense variants (c.319 T > C, Tyr107His; c.347C > T, p.Pro116Leu) in the CFAP410 gene. The authors found thinning and atrophy of the outer retina and reduced ERG responses. They also checked the protein stability infected with CFAP410 wild type and mutant proteins in cell culture and found reduced stability of the two mutant proteins, which might be associated with the ubiquitin-proteasome pathway.

Liu Y. et al. report the results from the application of dual gene therapy in a rd12 mouse model of RPE65 Leber congenital amaurosis. The subretinal application of vectors containing two genes: the hRPE65 gene and the Bcl-2L10 anti-apoptotic gene at postnatal day 14 resulted in preservation of outer nuclear layer thickness at 6 and 12 months after treatment. Additionally, a relatively preserved visual function (assessed by ERG) was maintained and some restoration was observed in the levels of the RPE65 protein and rhodopsin.

Wu et al. conducted a bioinformatics and machine learning study with the goal to investigate datasets related to non-specific orbital inflammation (NSOI), focusing on biomarkers and pathways central to the disease. After applying this approach, they established essential gene signatures related to this condition in 15 gene hubs (sets of genes interacting with each other) with 113 differentially expressed genes. Further, they found 9 miRNAs and 27 lncRNAs associated with NSOI. This study enhances our understanding of the pathogenesis of NSOI and points to future empirical avenues of inquiry to validate the findings.

Xia and Guo provided a mini-review on the topic of adeno-associated virus vectors for retinal gene therapy. They provide a concise summary of the many advancements in this field, focusing on AAV vectors for gene transfer into important ocular cell types, including RPE cells, photoreceptors, retinal ganglion cells, Müller cells, etc., via distinct injection methods. Thus, the authors highlight the progress and unmet needs of AAV vectors in retinal gene therapy.

Hua et al. explored a possible association between visceral fat area (VFA) and diabetic retinopathy (DR) among people with type 2 diabetes mellitus in a cross-sectional study involving 3,707 adult participants with type 2 diabetes mellitus in their 50 s, residing in Ningbo, China. Based on a regression model, they found a significant association between VFA and occurrence of DR, even after adjusting for various contributing factors and established that patients with high VFA levels had more retinopathy than those of normal VFA levels. They also found a modest yet statistically insignificant increase in VFA values as the severity of DR increases.

Finally, Seah et al. reviewed the current knowledge about modeling and potential treatment of inherited retinal diseases using human induced pluripotent stem cell derived photoreceptor cells and RPE cells. In their major review (113 references), they explored the considerations for developing disease models and discussed models for specific diseases, like retinitis pigmentosa, Leber's congenital amaurosis, choroideremia, gyrate atrophy, Stargardt disease, etc. They also reviewed technological hurdles and future directions in this space.

This Research Topic of articles indicates the substantial challenges that still exist in the diagnosis, understanding pathobiology, and development of novel, effective treatments for retinal neurodegenerative diseases. Only a comprehensive, multi-disciplinary approach would be successful in overcoming the various complexities and challenges that this type of diseases pose.

